# The greater effect of high-intensity interval training versus moderate-intensity continuous training on cardioprotection against ischemia-reperfusion injury through Klotho levels and attenuate of myocardial TRPC6 expression

**DOI:** 10.1186/s12872-019-1090-7

**Published:** 2019-05-16

**Authors:** Maral Ramez, Hamid Rajabi, Fatemeh Ramezani, Nasim Naderi, Amir Darbandi-Azar, Farinaz Nasirinezhad

**Affiliations:** 1 Department of Exercise physiology, Faculty of Physical Education and Sport Sciences, Kharazmi University, Tehran, Iran; 2 Physiology Research Center and Physiology Department, Faculty of Medicine, Iran University of Medical Sciences, Tehran, Iran; 3 Rajaie Cardiovascular Medical and Research Center, Iran University of Medical Sciences, Tehran, Iran

**Keywords:** Ischemia-reperfusion injury, Cardioprotection, Exercise training, Klotho, TRPC6

## Abstract

**Background:**

Myocardial ischemia-reperfusion (IR) injury is a leading cause of death all over the world, so developing practical approaches to promote cardioprotection against IR injury is essential. Exercise training is an effective strategy to improve cardioprotection. Hence, the purpose of this study was to investigate the effect of short-term preconditioning with two types of high-intensity interval training (HIIT) and moderate intensity continuous training (MICT) on klotho and TRPC6 mechanisms in cardioprotection.

**Methods:**

Eighty Male Wistar rats (250–300 g) were randomly divided into 7 groups, including Control, HIIT, MICT, Sham, IR, HIIT+IR, and MICT+IR. Training was performed in 5 consecutive days. HIIT protocol consisted of running on the treadmill at intervals 85–90% vo_2_max that separated by slow intensity periods at 50–60% vo_2_max. MICT program was performed at 70% VO_2_max at the same running distance with HIIT groups. The cardiac IR injury was induced by LAD occlusion followed by reperfusion. ELISA kit was used in order to measure the plasma levels of klotho, LDH and CK-MB, and TRPC6 expression was determined using the western blot technique. Data were analyzed using one way ANOVA and Tukey’s post hoc tests.

**Results:**

The results of this study showed that both types of exercise training programs significantly increase plasma levels of klotho and reduce the infarct size and heart injury. In addition, the exercise training decreased the amount of TRPC6 channels expression during IR. However, the effect of HIIT on increasing the klotho and cardioprotection was greater compared to MICT.

**Conclusions:**

Based on the results, even a short-term of aerobic exercise training, especially HIIT, promotes cardioprotection against IR injury and decreases infarct size via an increase in klotho and attenuate of protein expression of myocardial TRPC6 during IR.

## Background

Myocardial ischemia-reperfusion (IR) injury is a main and the pathological cause of morbidity and mortality during cardiovascular diseases, especially diseases associated with coronary artery, acute myocardial infarction, and even cardiac surgery and percutaneous coronary intervention [1, 2]. The levels of myocardial damage depend on the ischemic duration [3]. However, when the heart is regularly exposed to mild IR without cell death (such as conditions caused by exercise training), it leads to augment cardioprotection through the preconditioning process [4, 5]. In this regard, numerous studies show that regular exercise, even short-term of exercise training (1, 3 and 5 sessions) [4, 6–9], is one of the practical approaches of preconditioning to achieve cardioprotection and reduce the risk of IR-induced injury and death [4, 10]. However, despite the recognition of some effective mechanisms of Exercise-induced cardioprotection, the cellular and molecular mechanisms that induce this adaptation remain as a complex and debated issue [3]. Myocardial ischemia results in decreased cellular ATP, increased reactive oxygen species (ROS), accumulation of H^+^, elevated cytosolic calcium levels, and calpain activation. During reperfusion, production of ROS is exacerbated, cellular calcium overload increases and activation of calpain and caspase-3 and leukocyte infiltration occur [3]. Oxidative stress, ROS, mitochondrial permeability transition pore (mPTP) opening, and a disorder in calcium regulation are essential mechanisms in IR-induced injury, and inhibition of them plays a cardioprotective role [3, 11–14]. Calcium overload plays a critical role in cardiac injury and cell death in response to IR [15, 16]. The increased level of cytosolic calcium through various mechanisms, including activating calpain, facilitating production of mitochondrial ROS, mPTP opening, dissipate of mitochondrial membrane potential and further impairs ATP production, leads to mitochondrial dysfunction and increase cellular injury and cell death [17–19]. So it is likely that preventing calcium overload by the change in calcium channels and calcium handling proteins and improving the antioxidant status to be the main mechanisms of exercise-induced cardioprotection [3, 11, 12].

Evidence shows that the family of TRPC channels is involved in regulation of calcium entry into tissues and 6 members of this family have been identified in the heart [15, 20]. The physiological function of these channels under normal conditions is unknown, but stimulation of these channels in response to various stresses and increase of their function in different tissues (kidney, blood vessels, lung, and heart), lead to dysfunction and disease [21]. Despite studies that show the association between the IR injury and increase in TRPC6 expression in the lung [22] and kidney [23, 24], there are no reports that show this relationship in the heart [23], but it has been suggested that some TRPC subtypes may be associated with calcium-induced apoptosis in cardiomyocytes as a result of IR [15] and also, overexpression of TRPC6 are involved in cardiac pathological hypertrophy [21]. Therefore, due to the presence of these channels in the heart and their role in pathological hypertrophy, it seems that TRPC6 channels can be involved in response to IR injury in the heart too.

It has also been reported that the reduction in TRPC6 expression provides cardioprotection against pathological hypertrophy and soluble klotho can protect the heart via inhibiting TRPC6 channels [21, 25]. Klotho is an anti-aging protein that is mainly expressed in the kidney, brain, and pituitary glands, but has also been observed in many other tissues such as skeletal muscle, bladder, ovaries, testicles, thyroid gland, aorta, heart, and blood vessels (arteries) [25, 26]. The soluble and circulating form of klotho, which is the result of both cleavage from its transmembrane form and the secreted form of this protein, have biological effects on cells and organs of the body [26–28]. It is suggested that cardiac effects of Klotho may be due to inhibition of expression and exocytosis of TRPC6 channels [21, 25], suppression of cardiomyocyte apoptosis via downregulation of endoplasmic reticulum stress and a reduction in ROS production [28, 29]. However, changes in klotho and the role of this protein during myocardial IR are unknown and it is likely that the alterations of this protein and consequently its effect on expression of TRPC6 to be the mechanisms involved in the myocardial IR injury. On the other hand, based on limited studies that show the increase in soluble klotho following aerobic exercises [26, 30, 31], maybe an increase in klotho following exercise training or preventing its reduction during IR can reduce the heart’s susceptibility to injury. Therefore, the present study seeks to answer the question of whether Klotho and TRPC6 are involved in the mechanism of IR injury in the heart? And can a short-term of exercise training (HIIT and MICT) contribute to the reduction of IR injury by enhancing the Klotho protein and reducing TRPC6?

In general, due to the complexity of cardioprotection mechanisms, more research is needed to recognize mechanisms involved in this process and reduce the negative consequences of IR. Various studies have shown that the protective effects of exercise training are induced even after a short-term of exercise (1–5 sessions lasting from 30 to 60 min at 60–70%vo_2_max) [4, 6–9, 32], but unfortunately, there are still not many details about the proper intensity and type of aerobic training in order to improve cardioprotection. Moreover, most studies on cardioprotection against IR have focused on the effects of moderate intensity continuous training and there are limited studies about the effects of high-intensity interval training and its cardioprotection mechanisms [33]. Many studies show cardiovascular, muscular and metabolic adaptations are highly dependent on the intensity of training and the evidence recommended that exercise training intensity is the most critical factor to determine cardioprotection rather than duration [34–37]. Accordingly, HIIT which consists of repeated exercises at high-intensity at > 85–90% peak VO_2_ separated with periods of rest or low-intensity exercise for recovery, has received more attention currently [38]. Several studies have demonstrated that HIIT was more effective than MICT in inducing cardiovascular adaptations, peak oxygen uptake, ventricular function, cardiomyocyte contractility, endothelial function, and biomarkers associated with vascular function like oxidative stress, inflammation, and insulin resistance, in healthy individuals and patients with cardiovascular diseases [39–45]. So, it seems that the beneficial effects of preconditioning with HIIT on cardioprotection against IR may be greater than MICT, although studies on the effect of HIIT and its mechanisms of cardioprotection are very limited and more studies are needed. Therefore, due to the importance of selecting the correct and effective training program to improve cardioprotection, and also considering the different hemodynamic, physiological and metabolic effects of MICT and HIIT exercises, the purpose of this study was to investigate and compare the effect of preconditioning with short-term HIIT and MICT on plasma levels of klotho and myocardial TRPC6 expression as probable mechanisms of cardioprotection against IR injury.

## Methods

### Animals and experimental design

This study was performed according to guidelines for the care and use of laboratory animals published by the US National Institutes of Health (NIH Publication No.85–23, revised 1996). All experimental procedures (animal keeping, handling, exercise training, anesthesia, and sacrifice) were approved by the Animal Ethics Committee of University of Medical Sciences. In this study, 80 Male Wistar rats with a weight range of 250–300 g (8–10 weeks old) were purchased from the Pasteur Institute of Iran (Tehran, Iran) and kept in the standard environment (temperature of 22 ± 2 °C, relative humidity of 55%, and a dark-light cycle of 12:12 and free access to water and food). Animals were randomly divided into 7 groups, including control (*n* = 8), HIIT (*n* = 8), MICT (*n* = 8), Sham (*n* = 14), IR (*n* = 14), HIIT+IR (*n* = 14), and MICT+IR (*n* = 14). After training period and reperfusion phase, the rats were anesthetized with thiopental sodium (60 mg/kg i.p.) and the heart was immediately harvested and stored at − 80 °C until use.

### Exercise protocols

After a familiarization period (3 days) on the treadmill at a gradual increase in running times and speeds, the rats performed VO_2_max test based on the protocol of previous studies [45, 46]. Then, after 72 h, the animals performed 5 consecutive days of exercise Training. HIIT sessions included 5 min warm up (40–50%Vo_2_max), 6 × 2 min high intensity periods at 85–90% vo_2_max separated by 5 × 2 min periods of slow intensity at 50–60% vo_2_max (active recovery), and 5 min cooldown (40–50%Vo_2_max). The MICT program included 5 min warm up (40–50%Vo_2_max), then running on the treadmill at 70% VO_2_max that total running distance in each session was identical to the HIIT group (same running distance), and 5 min cooldown (40–50% Vo_2_max) (Table 1). The control group did not participate in any exercise program, but they were placed on the immobile treadmill to provide the same conditions for all subjects.

**Table 1:** Training protocol

	High intensity interval training (HIIT)	Moderate intensity continuous training (MICT)
Sessions (day)	5 consecutive days	5 consecutive days
Intervals	High intensity intervals 6 (6 × 2 min)	1 Continuous period with moderate intensity
	Low intensity intervals: 5 (5 × 2 min)	
Intensity (%vo_2_max)	High periods: 85–90% vo_2_max	70% vo_2_max
	Low periods: 50–60%vo_2_max	
Duration (min)	Warm up: 5 min	Warm up: 5 min
	HIIT: 22 min	MICT: 22–24 min
	Cooldown: 5 min	Cooldown: 5 min

### Myocardial ischemia-reperfusion injury

The rat model cardiac IR injury was performed by ligation of the left anterior descending coronary artery (LAD) for 30 min followed by 24 h reperfusion as previously described [47–49]. Briefly, after anesthesia (thiopental sodium; 60 mg/kg i.p.), the trachea was intubated and connected to a ventilator (Harvard rodent ventilator model 683, Holliston, MA, USA) and the heart was exposed through a left thoracotomy and pericardium incision. Then, the proline 0–6 polypropylene suture was passed approximately 1–2 mm distal from LAD artery origin and after the condition became stable, ischemia was induced for 30 min by ligation of LAD. Following LAD artery occlusion, the pale of the heart, ST elevation and electrocardiographic changes (ML750 PowerLab/4sp ADInstruments) confirmed successful ligation and myocardial ischemia. Rats in the sham group underwent the same surgical procedure without LAD ligation.

### Assessment of infarct size

Evans Blue-TTC staining was used to assess the infarct size according to the methods previously described [47–49]. At the end of reperfusion, the LAD artery was re-occluded at the same position and 2 ml Evans Blue 2% dye (Sigma) was injected through the femoral vein to distinguish the non-risk area of the heart (blue-stained) from the area at risk (AAR) (*n* = 6 from each group of Sham, IR, HIIT+IR, and MICT+IR). After that, the hearts were removed quickly, frozen and sliced into 2 mm transverse sections from apex to base. The slices were incubated in 1% 2, 3, 5 triphenyltetrazolium chloride (TTC in 0.1 M phosphate buffer, pH 7.4 Sigma) solution for 20 min at 37^0^ C and then fixed in 10% formalin for 24 h. Finally, the samples were scanned and the ischemic area (white un-stained) and area at risk were analyzed by Image J software. The area at risk was expressed as a percentage of left ventricle (AAR/LV) and infarct size was reported as a percentage of the area at risk (IS/AAR).

### Measurement of plasma levels of klotho, LDH, and CK-MB

24 h after the last training session, ischemia-reperfusion surgery was performed and after the completion of the reperfusion phase (24 h), blood samples were taken from the rat^,^s heart and poured into EDTA tubes to prevent clotting, and then, the plasma was separated by centrifugation. Plasma level of Klotho was evaluated by rat Klotho enzyme-linked immune Sorbent assay (ELISA) kit on the basis of the Biotin double antibody sandwich technology ((BT (bioassay technology laboratory), Shanghai crystal day biotech Co, LTD; Shanghai)). Markers of myocardial injury were determined by measuring the plasma levels of LDH and CK-MB using the standard ELISA kits (Pars Azmoon) and enzymes activity were presented in U/l. All procedures were performed according to the kits manufacturer’s instructions.

### TRPC6 protein expression

Western blot method was used to determine the changes in TRPC6 protein expression. The heart tissue (AAR of the left ventricle) was lysed and homogenized in RIPA Buffer (RIPA Lysis Buffer System, Santa Cruz Biotechnology; Wise Tis HG-15D Homogenizer). The total protein concentrations were determined by Bradford method and equal amount of protein samples was loaded in electrophoresis (Bio-Rad). Then, Protein samples were separated using 10% sodium dodecyl sulfate-Polyacrylamide gel electrophoresis (SDS-PAGE) and transferred to polyvinylidene fluoride membranes (PVDF; Amersham Hybond P) by Trans-Blot SD (Bio-Rad Semi-Dry Transfer Cell). The PVDF membranes were then incubated with primary TRPC6 antibody ((TRPC6 Polyclonal Antibody, Thermo Fisher Scientific, USA) and β-actin antibody as a loading control (Cell signaling Technology) followed by HRP-conjugated secondary antibody (Goat Anti Rabbit, Abcam). Eventually, The membrane was incubated with an ECL Western blotting system (Amersham ECL advance western blotting detection kit) and then exposed to X-ray film. Densitometry analysis of protein bands was performed using Image J software.

### Statistical analysis

Statistical analysis and investigation of differences between groups were performed by one-way analysis of variance (ANOVA) and Tukey post hoc tests. All data were analyzed using SPSS software version 16 (SPSS, Inc., Chicago, IL, USA). All values were presented as means ± SD and *P* < 0.05 was considered statistically significant.

## Results

**Fig. 1:**
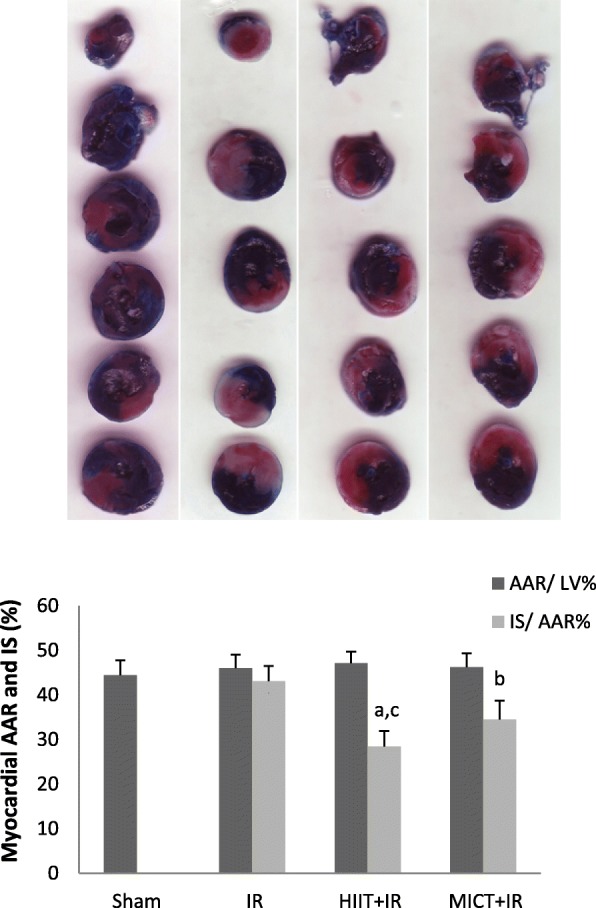
Myocardial area at risk (AAR/LV %) and Infarct size (IS/AAR %) following ischemia-reperfusion (*n* = 6). ^a^ *P* < 0.001, ^b^ *P* < 0.01 vs IR group, and ^c^ *P* < 0.05 vs MICT+IR group. There was no significant difference in AAR between the groups

**Fig. 2:**
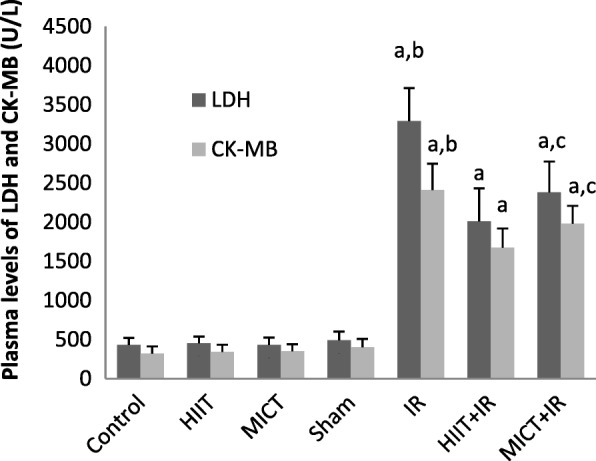
Plasma levels of LDH and CK-MB in experimental groups (*n* = 8). ^a^ *P* < 0.001 vs groups without IR, ^b^ *P* < 0.001 vs both Training + IR groups (HIIT+IR and MICT+IR), and ^c^ *P* < 0.05 vs HIIT+IR group

**Fig. 3:**
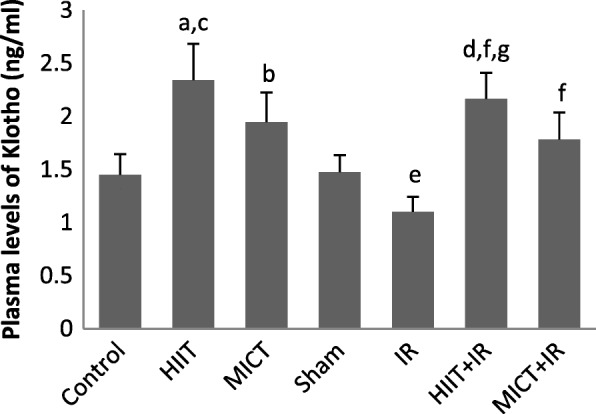
Plasma Klotho levels in experimental groups (*n* = 8). ^a^ *P* < 0.001 and ^b^ *P* < 0.01 compared to Control group, ^c^ *P* < 0. 05 vs MICT, ^d^ *P* < 0.001 and ^e^ *P* < 0.05 vs sham, ^f^ *P* < 0.001 vs IR, and ^g^*P* < 0.05 vs MICT+IR

**Fig. 4:**
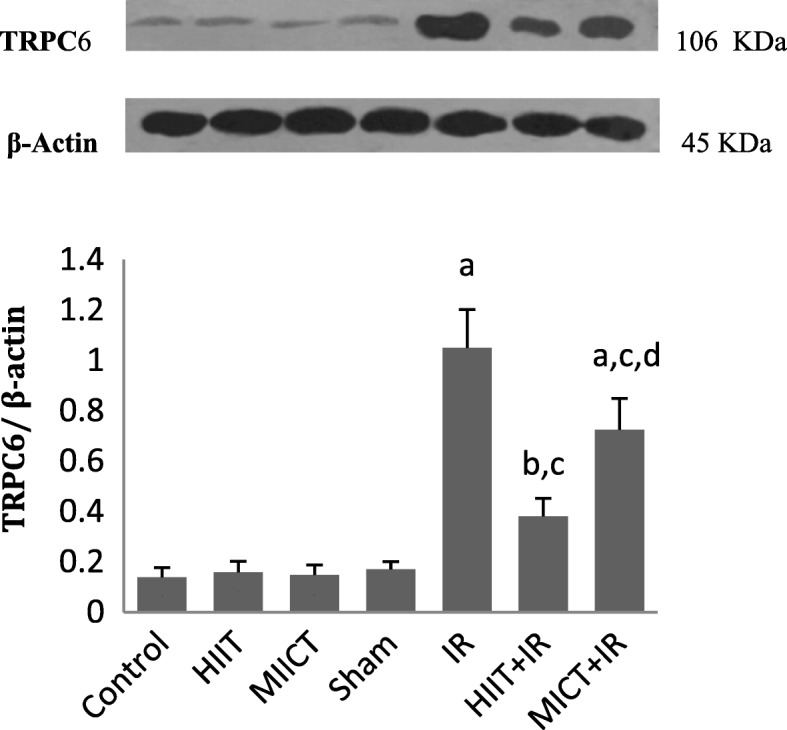
Ratio of TRPC6 to β-actin expression in experimental groups (*n* = 4). ^a^ *P* < 0.001 and ^b^ *P* < 0.05 vs Sham, ^c^ *P* < 0.001 vs IR, and ^d^ *P* < 0.001 vs HIIT+IR group

As shown in Table 2, there was no significant difference in body weight and heart weight between experimental groups (*P* > 0.05). Also, there were no statistically significant difference in vVo_2_max (Velocity at VO_2_max) and Vo_2_max between groups at the beginning of the training. Following IR injury, there was no significant difference in the size of the area at risk (AAR) (*P* = 0.497), indicating no difference in ischemic pattern and similar coronary blockade in different groups. As shown in the figure of TTC-stained (Fig. 1), the infarct size in IR group was 43.10 ± 3.35, but the results of this study indicated that 5 consecutive days of aerobic exercise training with both high-intensity interval training and moderate-intensity continuous training decreased infarct size in rats (*p* < 0.001; Fig. 1). The results indicated that the effect of HIIT on reducing the infarct size was higher than the MICT (34 and 20%, respectively). The significant elevated levels of CK-MB and LDH were found after IR injury, but levels of these cardiac injury markers after IR injury were also lower in training groups compared to the IR group (*p* < 0.001; Fig. 2). Moreover, the infarct size and enzyms levels in the HIIT group were significantly less than MICT (*P* < 0.05), which indicated a greater impact of high-intensity interval training program compared to the MICT in cardioprotection. The obtained data showed a significant increase in plasma levels of Klotho following both exercise training protocols (HIIT, and MICT), that it was significantly higher in HIIT than MICT group (*P* = 0.028). The amount of this protein decreased following ischemia-reperfusion in the IR group compared to the sham group (*P* = 0.046), but no significant change was observed in the training groups. The amount of klotho in the training groups following IR (HIIT+IR and MICT+IR) was higher than IR (*P* < 0.001; Fig. 3), which may reflect the effect of exercise training-induced Klotho enhancement, especially by HIIT, in protection from heart against IR. Reducing Klotho levels following injury in training groups was less than the untrained group (IR) group and was not significant, suggesting that the exercise training prevented a significant reduction in Klotho following IR injury. The comparison of TRPC6 expression between the groups showed that there was no significant difference between uninjured groups, but IR injury increased expression of these channels. The expression of TPPC6 was increased following IR, but this increase was lower in training groups (especially HIIT) compared to the IR (*p* < 0.001). The results of TRPC6 expression are shown in Fig. 4.

**Table 2:** Body weight, heart weight, Vo_2_max and vVo_2_max of rats in experimental groups

	Control	HIIT	MICT	Sham	IR	HIIT+IR	MICT+IR	*p*
	Means ± SD	Means ± SD	Means ± SD	Means ± SD	Means ± SD	Means ± SD	Means ± SD	
Body Weight (g)	282.57 ± 13.04	275.12 ± 14.31	278.53 ± 13.37	277.67 ± 15.10	290.12 ± 12.35	285.33 ± 12.15	288.54 ± 10.04	0.183
Heart weight (g)	1.30 ± 0.10	1.20 ± 0.16	1.28 ± 0.16	1.27 ± 0.20	1.34 ± 0.11	1.28 ± 0.11	1.38 ± 0.088	0.589
vVo_2_max (m/min)	42.87 ± 1.24	42.62 ± 1.40	43.12 ± 1.35	44.12 ± 1.24	43.50 ± 1.19	42.75 ± 1.38	42.50 ± 1.60	0.224
Vo_2_max (ml/kg^0.75^ per min)	114.70 ± 3.36	114.04 ± 3.80	115.39 ± 3.66	118.09 ± 3.35	116.40 ± 3.23	113. 02 ± 3.75	113.69 ± 4.34	0.115

## Discussion

The results of this study show that even a short-term of aerobic exercise training by both high-intensity interval training (HIIT) and moderate-intensity continuous training (MICT) induces cardioprotection against IR and reduces the infarct size by approximately 34 and 20%, respectively, compared with the IR group. Also, lower plasma levels of CK-MB and LDH following IR in training groups (HIIT+IR and MICT+IR) compared to the IR confirm the exercise training cardioprotective effects in reducing infarct size*.* Moreover, the significant differences in infarct size and markers of cardiac injury between these two training groups show a greater influence of HIIT than MICT on cardioprotection. It seems that the duration of the training period is not a limiting factor for exercise-induced cardioprotection, because in line with the results of this study, the reduction of infarct size and the effects of cardioprotection against IR injury have been reported even after a short-term exercise training (1 to 5 days), Although this protection surely augments with longer training periods [4, 6–9, 32]. This means that, although the short-term of exercise training does not cause structural changes in the heart, it can induce changes in cardiac phenotype and cellular-molecular adaptations that resist IR-induced myocardial injury [3, 33]. Despite studies that show the impact of preconditioning with moderate-intensity continuous training on cardioprotection, research about the effects of High-intensity interval training and its mechanisms is very limited [33, 50]. Since the intensity of exercise training is a critical factor in cardioprotection, the present study has investigated and compared the impact of preconditioning with these two types of exercise training on cardioprotection with regard to the klotho and TRPC6 channels axis for the first time. Comparing these two exercise training protocols (HIIT and MICT) can play an important role in identifying a more effective training program on cardioprotection. The mechanisms responsible for cardioprotection following exercise training remains a debated issue because of numerous affecting factors, including an increase in antioxidant capacity and levels of HPSs, alteration in glycolytic flux and nitric oxide (NO) signaling, improvement of ATP-dependent potassium channel function, augmentation of myocardial COX-2 activity, elevation of endoplasmic reticulum (ER) stress proteins, and changes in mitochondrial phenotype [3, 8, 10–12]. In sum, despite some known cellular and molecular mechanisms in exercise-induced cardioprotection, all mechanisms responsible for cardiovascular adaptations and increased cardioprotection following exercise training, especially HIIT, are largely unknown and more research is needed to identify these mechanisms. The results of this study illuminate and explain novel mechanisms and molecular pathway (Klotho-TRPC6 axis (in Exercise-induced cardioprotection (EICP). Understanding the molecular mechanisms of EICP leads to the development of applied approaches to prevent myocardial IR injury and even a safe and effective treatment.

Although the physiological function of TRPC6 channels in the heart in normal conditions remains poorly understood, it has been reported that abnormal influx calcium through TRPC6 channels in response to different types of stress such as increased ROS, PLC and DAG activation, Excessive stimulation by endothelin1, angiotensin II, and vasoconstriction activates calcineurin and NFATs and leads to pathological hypertrophy and heart failure [13, 21]. In addition, the activity and upregulation of these channels provide a feedback loop that activates calcineurin/NFAT-mediated TRPC6 transcription and increases the expression and exocytosis of these channels and amplifies the pathological responses [21]. The results of this study show the association, changes, and the role of TRPC6 channels in cardiac IR injury and provide new evidence from potential mechanisms of ischemia-reperfusion injury. Investigating and recognizing the mechanisms and molecular pathways involved in IR injury can help develop preventive and therapeutic strategies. In the present study, increased TRPC6 expression following IR injury suggests that these channels can be involved in response to IR and mechanisms of injury in the heart. Along with our results, Xiju He et al. also reported that Blocking TRPC activity with SKF96365 or lacking of these channels with genetic ablation in mice protects against H/R and I/R injury [14]. Therefore, our findings implicate that IR injury and increased oxidative stress can activate and enhance the expression of these channels that lead to increase calcium entry and ultimately enhance cardiac damage. The lower increase in TRPC6 expression in training groups following IR and oxidative stress compared to the IR group can be considered as one of the EICP mechanisms. Moreover, lower TRPC6 expression in the HIIT+IR group compared to the MICT+IR group can also reflect the greater effect of HIIT on cardioprotection and oxidative stress tolerance via this mechanism. However, more studies are needed to clarify this effect and its mechanisms. On the other hand, reports show that decrease in TRPC6 expression or inhibition of them by gene silencing or by dominant-negative expression of mutant channels decreases cardiac sensitivity to stress and provides cardioprotection against pathologic hypertrophy [21]. It has been shown that soluble klotho can inhibit these channels [21]. Cardiac effects of soluble klotho may be due to inhibition of expression and exocytosis of TRPC6 channels, suppression of cardiomyocyte apoptosis via downregulation of endoplasmic reticulum stress and reducing the ROS production [21, 28, 29]. Our findings show changes of klotho and the role of this protein during myocardial IR and also its changes following two different forms of training, especially HIIT (for the first time). In our study, an increase in Klotho levels following exercise training, especially HIIT, and lower expression of TRPC6 channels during IR in training groups compared to the IR group, maybe confirm the role of soluble Klotho in inhibiting TRPC6 channel expression and less injury. Klotho and its protective effect against IR can be considered as a preventive and therapeutic factor for cardiovascular disease and attenuating factor of ischemic-reperfusion injury, which can be increased by exercise training. Due to the limited studies about the effect of exercise training on Klotho protein, the underlying mechanisms explaining the increase in soluble Klotho following exercise training have not yet been elucidated [26, 30]. But it is possible that aerobic exercise training increases the soluble Klotho concentration through the intervention in upregulation of the secreted form of Klotho or an increase in the extracellular domain shedding of its membrane form [30]. However, it is not yet clear whether muscle contraction increases the expression and muscular production of this protein, or some myokines during exercise training are responsible for increasing klotho expression and releasing it into the bloodstream from other tissues such as the kidneys and the brain [26]. Various studies have shown that exercise training increases transcription factor of Peroxisome proliferator-activated receptor γ (PPARγ), NO production, and antioxidant capacity, and also decreases oxidative stress, angiotensin II type I receptor (AT1R), ET1, TGFβ, and inflammation [30, 51–55]. Since these factors affect the expression of klotho mRNA and protein [30, 56–59], therefore, the increase in circulating Klotho following exercise training could be the result of these changes. Also, the cause of the significant difference in klotho levels between training groups (HIIT and MICT) might be different effects of these two types of exercise training on these variables. It is likely that more stimulation of PGC1α and PPAR-γ expression and maybe more hypoxia following HIIT to be a larger stimulus to increase the Klotho compared to MICT, although these probabilities need to be investigated. Moreover, different cardiovascular, muscular and metabolic responses and adaptations of these two protocols and also different patterns in shear stress, changes in vasodilation and endothelial biomarkers and antioxidant capacity following exercise training can also be considered as mechanisms of more prominent changes in klotho levels following HIIT than MICT. Anyway, further studies and different training protocols should be investigated to determine the effective mechanisms for exercise training-induced changes in Klotho levels. On the other, the results of this study indicate a decrease in plasma levels of Klotho following IR injury in IR group, but responsible mechanisms of this change are unknown and more research is required to identify and corroborate it. Nevertheless, this may be explained by increased oxidative stress and ROS during IR that can decrease expression and synthesis of Klotho in various tissues [57] such as kidney and brain, and maybe in the heart and aorta and blood vessels, and its cleavage into the bloodstream. The studies show that It is possible that the increase in TNFα, IFNγ, and inflammation following IR also reduce the production and release of Klotho. Though, the role of TNFα and IFNγ in decreased expression of klotho during IR is unknown [60, 61]. It has been shown that acute kidney injury (AKI) is a state of transient Klotho deficiency after most cardiac surgeries [62], hence Myocardial IR-induced AKI could also be responsible for Klotho reduction that requires further investigation. This reduction in klotho following IR may also contribute to augment cardiac injury through affecting on TRPC6 channels and antioxidant defense. So, according to the results of this study, the increase in soluble Klotho following exercise training and the prevention of significant decline in this protein during IR can reduce the susceptibility of the heart to oxidative stress and IR injury. However, more research is needed to find out the effective mechanisms in Klotho alteration following different types of exercise training and the role of this protein in cardioprotection. Understanding the role of exercise training and skeletal muscles as a regulator of Klotho expression is important and can lead to the development of cardiovascular health and prevention and rehabilitation programs.

## Conclusion

The present study indicates that even a short-term of aerobic exercise training (both HIIT and MICT) protects the heart against the ischemia-reperfusion injury and reduces the infarct size. According to the findings of this study, the increase in TRPC6 expression and decrease in Klotho following IR could be as mechanisms involved in the cardiac IR injury and be considered as therapeutic and prevention targets. On the other, increased plasma levels of Klotho as a result of exercise training and prevention of significant reduction in klotho during IR, and consequently, the lower expression of TRPC6 can enhance cardioprotection and decrease cardiac injury. Moreover, the results of this study show the superior influence of HIIT on cardioprotection than MICT through these mechanisms. However, more studies are needed to clarify this effect and mechanisms that might influence it.

## Abbreviations

AKI: Acute kidney injury
AT1R: Angiotensin II type I receptor
COX-2: Cyclooxygenase-2
ELISA: Enzyme-linked immune sorbent assay
ET1: Endothelin 1
HIIT: High-intensity interval training
HPSs: Heat shock proteins
IFNγ: Interferon gamma
IR: Ischemia-reperfusion
MICT: Moderate intensity continuous training
NFAT: Nuclear factor of activated T-cells
NO: Nitric oxide
PPARγ: Peroxisome proliferator-activated receptor γ
PGC1α: Peroxisome proliferator-activated receptor gamma coactivator 1-alpha
ROS: Reactive oxygen species
TGFβ: Transforming growth factor beta
TNFα: Tumor necrosis factor-α
TRPC: Transient receptor potential cation channels
